# Development of microsatellite markers for the resin‐yielding, non‐timber forest product species *Boswellia serrata* (Burseraceae)

**DOI:** 10.1002/aps3.1180

**Published:** 2018-09-19

**Authors:** Bhavani Shankar Maradani, Ravikanth Gudasalamani, Siddappa Setty, Rajasekaran Chandrasekaran

**Affiliations:** ^1^ Ashoka Trust for Research in Ecology and the Environment (ATREE) Royal Enclave, Srirampura, Jakkur PO Bangalore 560064 India; ^2^ School of Bio Sciences and Technology Vellore Institute of Technology University Vellore 632014 India

**Keywords:** aromatic resin, *Boswellia serrata*, Burseraceae, microsatellites, non‐timber forest product (NTFP) species

## Abstract

**Premise of the Study:**

*Boswellia serrata* (Burseraceae) is an economically important aromatic, gum‐resin–yielding, non‐timber forest tree species. Microsatellite markers were developed for *B. serrata* for the first time to study genetic diversity and population structure.

**Methods and Results:**

A magnetic bead enrichment method was used to develop 16 microsatellite markers, of which 11 were polymorphic. The number of alleles per locus in the 60 individuals studied ranged from three to 10, and the levels of observed and expected heterozygosity ranged from 0.50 to 0.90 and 0.666 to 0.861, respectively. The primers successfully amplified in the congeneric species *B. ovalifoliolata*.

**Conclusions:**

These microsatellite markers can be used to study the genetic variation and population structure of *B. serrata* and to provide crucial information on population and ecological issues for management and conservation of the species.

Natural gums are one of the important non‐timber forest products (NTFP) in India. India is endowed with highly diverse gum‐yielding tree species and is a leading producer of natural gums (Basch et al., 2004). Olibanum, an oleo‐gum‐resin, is obtained from the bark of *Boswellia* Roxb. ex Colebr. species (Burseraceae). *Boswellia serrata* Roxb. ex Colebr. (Leung and Foster, 1996), one of four species in the genus *Boswellia*, is an endangered species that is found in dry deciduous forests of India, Pakistan, and Arabia (Ghorpade et al., 2010). It is often referred to as Indian frankincense and is locally called dhoopa or salai guggal. *Boswellia serrata *is a moderate‐ to large‐sized tree found in the deciduous forests of Western Ghats, Eastern Ghats, Rajasthan, Gujarat, Uttar Pradesh, and in other dry and tropical regions of India. Burning *B. serrata* resin as incense has been part of religious and cultural ceremonies since time immemorial. *Boswellia serrata* gum‐resin contains essential oils, volatile oils, sugars, and terpenes with β‐boswellic acid (Siddiqui, 2011). In recent years, *B. serrata* has attracted the attention of pharmacologists for the development of nonsteroidal anti‐inflammatory drugs (NSAIDs) because of its chemical constituents (Singh et al., 1996). Oleo‐gum‐resin of *B. serrata* is used in the treatment of ulcerative colitis, Crohn's disease, osteoarthritis, rheumatoid arthritis, joint pain (rheumatism), bursitis, abdominal pain, asthma, hay fever, sore throat, syphilis, and liver disorders; it can act as an anti‐inflammatory agent by inhibiting the synthesis of 5‐lipoxygenase (Siddiqui, 2011). *Boswellia serrata* extract also exhibits antibacterial, antifungal, and antimicrobial activities (Ismail et al., 2014). In recent years, it has also been used in cosmetics and perfumes.

In India, approximately 1500 tons of *B. serrata* gum is harvested annually (Giri et al., 2008). With increasing demand for resin, unsustainable harvesting practices, anthropogenic threats, and lack of regeneration (Tandon et al., 2010) have resulted in the rapid decline of populations of the species. Extraction of the resin by puncturing or damaging the bark can cause trees to be susceptible to pests or diseases, and indiscriminate extraction could lead to mortality. This adult mortality, in combination with fragmentation and low regeneration, threatens the persistence of the species. *Boswellia serrata* is now reported as a rare species in the *Red Data Book of India* (Modi and Mathad, 2016). Despite its economic importance, knowledge of the genetic resources of this species is scarce. Information on the genetic diversity and pattern of genetic differentiation across populations is crucial for designing appropriate conservation strategies. Addisalem et al. (2015) developed microsatellite markers for *B. papyrifera*. However, when we used these markers in *B. serrata*, we found their amplification efficiency to be low. In this study, we developed microsatellite markers for *B. serrata* with high efficiency and tested their applicability in the related species *B. ovalifoliolata* N. P. Balakr. & A. N. Henry.

## Methods and results

### Sample collection and DNA extraction

Fresh leaves of *B. serrata* were collected from three populations, Biligiri Ranganathaswamy Temple Tiger Reserve (BRT‐TR), Male Mahadeshwara Hills Wildlife Sanctuary (MM Hills WLS), and Cauvery Wildlife Sanctuary (Cauvery WLS) of Western Ghats, India, to develop genetic markers and from one population of *B. ovalifoliolata*, from the Seshachalam foothills of Tirupati, Andhra Pradesh, India, to check cross‐amplification (Appendix 1). Genomic DNA was extracted from leaf material using a cetyltrimethylammonium bromide (CTAB) method (Sambrook et al., 1989).

### Microsatellite library construction and primer design

A microsatellite enrichment library was constructed using a magnetic bead hybridization method following Glenn and Schable (2005) with minor modifications. Total genomic DNA of one sample from BRT‐TR was digested with the restriction enzymes *Rsa*I and *Xmn*I (New England Biolabs, Ipswich, Massachusetts, USA). Digested products were ligated to double‐stranded SNX linkers using a rapid DNA ligation kit (Fermentas International, Thermo Fisher Scientific, Bangalore, India) and amplified with SNX primers. The amplified products were hybridized with 3′‐biotinylated microsatellite probes, and hybridized probes were captured by streptavidin‐coupled (M‐280) Dynabeads (Invitrogen, Oslo, Norway). The captured fragments containing microsatellite repeats were enriched by amplification with SNX linker‐specific primers. Enriched fragments were transformed into *E. coli* strain CB‐5α with the pTZ5RT vector (Thermo Fisher Scientific, Bangalore, India). Recombinant clones were identified by colony PCR using M13 primers. The PCR fragments larger than 300 bp were sequenced using an ABI PRISM 3130 Genetic Analyzer (Applied Biosystems, Waltham, Massachusetts, USA) at Chromous Biotech (Bangalore, India). After trimming vector and linker sequences, 216 nonredundant contig sequences were obtained, of which 68 contained microsatellite repeats. A total of 42 primer pairs were designed using the software Primer3Plus (Untergasser et al., 2007) with the following criteria: amplicon size 100–300 bp, annealing temperature 52–60°C, and GC content 40–60%.

### Validation and evaluation of designed markers

Of the 42 primer pairs, 16 successfully amplified (Table 1) and were tested for polymorphism in 60 individuals of *B. serrata* and for cross‐amplification in 10 individuals of *B. ovalifoliolata* (Table 2). PCR reactions were performed in a 15‐μL reaction volume containing 10–20 ng of template DNA, 1 mM of dNTP mix, 1× polymerase buffer, 1 unit of *Taq* polymerase (all reagents from Bangalore Genei, Bangalore, India), and 0.2 μM of each primer. The PCR profile was as follows: an initial denaturation at 95°C for 5 min; followed by 35 cycles of 95°C for 30 s, 50–60°C for 30 s, and 72°C for 30 s; and a final extension at 72°C for 10 min. Amplicons were analyzed on an ABI 3730 Genetic Analyzer (Applied Biosystems) at Chromous Biotech (Bangalore, India) and scored using GeneMapper version 3.2 (Applied Biosystems) software.

**Table 1:** Characteristics of 16 microsatellite markers developed in *Boswellia serrata*

Locus	Primer sequences (5′–3′)	Repeat motif	Fluorescent label	*T*_a_ (°C)	Allele size range (bp)	GenBank accession no.
BS6	F: CTACGTATTGATGAGGCGGC	(GA)_14_	PET	60	172–228	MG811526
	R: GAGATCGATGGAATTGCTGGT					
BS8	F: CGCCTCAGCTCACCAGTAAT	(CAAAA)_4_…(CAA)_4_	PET	52	173–209	MG811527
	R: CTGCCAGAGTATGAGAAGCAA					
BS10^a^	F: ACACGGGTCTGAACTCCAAC	(AAG)_20_	NED	60	205	MG811542
	R: GGATGATTCATTCCTGGAAAA					
BS11	F: AACAACCAACCCATCTCACTC	(TC)_7_	NED	54	172–206	MG811528
	R: GGTCGGTTGAGGATGGAATA					
BS12^a^	F: ATCGGGTGATTCTGCTATCG	(AG)_9_	HEX	58	174	MG811537
	R: GCAGAATCGCTACTCGATGA					
BS13	F: TTCCTTGGACATAGCCAAATCT	(AG)_11_	6‐FAM	58	258–296	MG811529
	R: GTAGACCTAGCATCTTCAGCTG					
BS14	F: CCGGCGCAACTTCAATAATA	(AAC)_4_	6‐FAM	58	183–208	MG811530
	R: ATCAGCAAGTCCGTCTGTCC					
BS16	F: CGCTTTCTATTTTCCTTTTTGG	(CT)_15_	HEX	50	118–138	MG811531
	R: GCTAACGATTGACAACTGCTG					
BS18^a^	F: CAACGAGAGGAGGCAGTGAT	(TC)_30_	6‐FAM	58	198	MG811540
	R: TTAAGGCCTGGCTAGCAGAA					
BS19^a^	F: GGATCCAGCCGCAGTATATC	(TG)_32_	HEX	56	224	MG811541
	R: TCGACAGCTCAAGGAATGTG					
BS21^a^	F: CAGCCTTCCTCAATCGGATA	(TGT)_12_	NED	52	218	MG811539
	R: AAGTCGGTCACCTCATTGGA					
BS23	F: CACGATGACGTGATTCTGCT	(CA)_9_	NED	52	188–216	MG811532
	R: CAAGCTTGCACACAGGAAAA					
BS25	F: TCAAGCCGTTGTAGTTGGTG	(TC)_15_	6‐FAM	52	206–252	MG811533
	R: TGGAAGCACAGAAAGAAGCA					
BS28	F: CCAGCATTTTTCCTTTCTTTTT	(AAG)_13_	PET	56	156–185	MG811538
	R: TTGCTCACGAAATCCTTCCT					
BS29	F: CACGATGACGTGATTCTGCT	(AG)_9_	HEX	52	184–228	MG811535
	R: CTTACACCATCTCCCTCTGC					
BS32	F: CTGCCAGGCCTTAAACAAAA	(TG)_17_	NED	60	242–270	MG811536
	R: GCAGTGGATGGGGTAGAATC					

Note

*T*_a_ = annealing temperature.

a Monomorphic locus.

**Table 2:** Genetic analysis of 11 polymorphic microsatellite markers developed for *Boswellia serrata* and cross‐amplification to *B. ovalifoliolata*.^a^

Locus	*Boswellia serrata*												*Boswellia ovalifoliolata*			
	BRT‐TR (*N* = 20)				MM Hills WLS (*N* = 20)				Cauvery WLS (*N* = 20)				Seshachalam Hills (*N* = 10)			
	*A*	*H*_o_	*H*_e_ b	PIC	*A*	*H*_o_	*H*_e_ b	PIC	*A*	*H*_o_	*H*_e_ b	PIC	*A*	*H*_o_	*H*_e_ b	PIC
BS6	7	0.75	0.838^ns^	0.817	8	0.75	0.851^ns^	0.834	8	0.65	0.853^ns^	0.836	5	0.7	0.76^ns^	0.720
BS8	3	0.55	0.666^ns^	0.592	3	0.55	0.666^ns^	0.592	3	0.5	0.666^ns^	0.592	3	0.5	0.655^ns^	0.580
BS11	5	0.7	0.778^ns^	0.743	5	0.7	0.79^ns^	0.757	7	0.6	0.808*	0.783	6	0.8	0.725^ns^	0.683
BS13	9	0.75	0.851^ns^	0.835	8	0.85	0.855^ns^	0.838	7	0.8	0.843^ns^	0.824	9	0.8	0.75^ns^	0.729
BS14	5	0.7	0.795^ns^	0.762	5	0.7	0.797^ns^	0.765	4	0.8	0.728^ns^	0.679	5	0.6	0.77^ns^	0.730
BS16	7	0.75	0.838^ns^	0.817	8	0.75	0.851^ns^	0.834	8	0.7	0.852^ns^	0.834	5	0.7	0.76^ns^	0.720
BS23	10	0.75	0.857^ns^	0.841	8	0.8	0.842^ns^	0.823	10	0.75	0.861^ns^	0.845	6	0.8	0.755^ns^	0.718
BS25	6	0.75	0.801^ns^	0.771	7	0.75	0.833*	0.812	7	0.75	0.837**	0.817	6	0.8	0.8^ns^	0.772
BS28	6	0.65	0.802^ns^	0.772	6	0.9	0.81^ns^	0.784	4	0.8	0.728^ns^	0.679	5	0.6	0.77^ns^	0.730
BS29	10	0.85	0.837^ns^	0.819	6	0.75	0.831^ns^	0.808	6	0.75	0.831^ns^	0.808	8	0.9	0.78^ns^	0.758
BS32	10	0.7	0.851^ns^	0.834	6	0.55	0.797^ns^	0.766	10	0.65	0.846^ns^	0.828	7	0.7	0.83^ns^	0.808
Mean (SD)		0.718 (±0.075)	0.81 (±0.054)	0.782 (±0.071)		0.731 (±0.107)	0.811 (±0.053)	0.783 (±0.07)		0.704 (±0.096)	0.805 (±0.066)	0.775 (±0.084)		0.718 (±0.116)	0.759 (±0.044)	0.722 (±0.057)

Note

*A* = number of alleles; *H*_e_ = expected heterozygosity; *H*_o_ = observed heterozygosity; *N* = number of individuals sampled; PIC = polymorphism information content.

a Locality and voucher information are available in Appendix 1.

b Deviation from Hardy–Weinberg equilibrium: *P < 0.05, **P < 0.01, ns = not significant.

Population genetic diversity parameters, i.e., observed heterozygosity (*H*_o_), expected heterozygosity (*H*_e_), number of alleles per locus (*A*), effective number of alleles (*A*_e_), Shannon's information index, and the probability of deviations from Hardy–Weinberg equilibrium were estimated using GenAlEx 6.5 (Peakall and Smouse, 2012). Polymorphism information content (PIC) and probability of identity (PI) were calculated by CERVUS 3.0 (Kalinowski et al., 2007).

Of the 16 successfully amplified primers, 11 were found to be polymorphic, of which eight had dinucleotide, two had trinucleotide, and one had compound repeat motifs (Table 1). Significant differences were found in allele frequencies between the analyzed populations. *A* ranged from three to 10, and levels of *H*_o_ and *H*_e_ of each population ranged from 0.50 to 0.90 and 0.666 to 0.861 (Table 2), respectively. Shannon's information index values ranged from 1.098 to 2.072 and PIC ranged from 0.592 to 0.845 (Table 2), indicating that the markers designed are highly polymorphic (PIC > 0.5) and informative. The PI value is low for many loci, with the combined PI value of 2.081E‐0015 confirming their applicability for population genetic studies. Significant deviations (*P* < 0.05 and *P* < 0.01) from Hardy–Weinberg equilibrium were detected for two markers (BS11 and BS25) but were not consistent across the populations. In *B. ovalifoliolata*,* A* ranged from three to nine, *H*_o_ ranged from 0.5 to 0.9, and *H*_e_ ranged from 0.66 to 0.83 (Table 2). Raw genotyping data for both species are available in Appendix S1.

## Conclusions

In this study, 16 microsatellite markers were developed specifically for *B. serrata* and 11 of these showed considerable polymorphism in all three studied populations. These markers will be useful for studying genetic diversity, gene flow, population structure, and inbreeding. The resulting information will help in developing appropriate strategies for sustainable utilization and conservation of this important resin‐yielding tree. The allelic overlap and intrageneric amplification of these microsatellite markers indicate a close relationship between *B. serrata* and *B. ovalifoliolata*, which needs support from further investigation.

## Data accessibility

Sequence information for the developed primers has been deposited to the National Center for Biotechnology Information (NCBI); GenBank accession numbers are provided in Table 1. Raw genotyping data for both species are available in Appendix S1.

## Supporting information


**APPENDIX S1.** Genotyping data for Boswellia serrata and B. ovalifoliolata.

## Voucher information for *Boswellia* species used in this study.

**Table:**

Species	Voucher specimen accession no.^a^	Collection locality (Code)	Geographic coordinates	*n*
*Boswellia serrata* Roxb. ex Colebr.	ATREEBs5925a	Biligiri Rangaswamy Temple Tiger Reserve (BRT‐TR)	11°59′38″N, 77°8′26″E	20
*B. serrata*	ATREEBs5925b	Male Mahadeshwara Hills Wildlife Sanctuary (MM Hills WLS)	12°1′50.52″N, 77°35′16.8″E	20
*B. serrata*	ATREEBs5925c	Cauvery Wildlife Sanctuary (Cauvery WLS)	12°10′12″N, 77°32′34.8″E	20
*B. ovalifoliolata* N. P. Balakr. & A. N. Henry	ATREEBo5926a	Seshachalam Hills	14°19′59.99″N, 78°15′0″E	10

Notes

*n* = number of individuals sampled.

a One sample per population is deposited at the ATREE Herbarium, Bangalore, India.
